# Hospital and Institutional News

**Published:** 1914-08-15

**Authors:** 


### HOSPITAL AND INSTITUTIONAL NEWS.

### GIFT OF A BASE HOSPITAL TO THE QUEEN: ARRANGEMENTS AND STAFF.

The Queen and Princess Christian have been offered and have accepted the gift of a fully-equipped Base hospital, which they have given to the Navy for the reception and accommodation of sick and wounded naval officers and men. The hospital, which will be erected at South Queensferry, in Scotland, will be built under the direction of Mr. Frederick W. Marks, F.R.I.B.A., by Messrs. Humphreys, Limited. The projected hospital has been entrusted to Mr. Alfred Mosely., C.M.G., to carry out; he served during the Boer War with the Princess Christian Hospital in South Africa. Mr. Mosely, who is a Knight of Grace of the Order of St. John of Jerusalem, has also had considerable organising experience in connection with the Industrial and Educational Commissions to America, and has written numerous reports on industrial and educational questions. The staff of the new hospital is to be drawn, with one exception, from the London Hospital, and comprises the following medical men:—Principal Surgeon.—Lieutenant-Colonel T. H. Openshaw, C.M.G., F.R.C.S. Surgeons.—Mr. A. B. Roxborough, F.R.C.S., and Mr. C. M. Kennedy, F.R.C.S. Anæsthetist and X-ray Expert.—Dr. A. L. Flemming, M.B., L.R.C.P., of Bristol. Assistant Surgeons.—Messrs. W. Ash, M.B., B.S., H. T. R. Moloney, M.R.C.S., L.R.C.P., and H. C. Billings, M.R.C.S., L.R.C.P. Dressers.—Messrs. L. G. Brown, G. D. Carr, J. N. Deacon, W. S. Herman, H. S. Jefferies, F. M. Mosely, G. T. C. Mosse, J. C. Russell, R. O. Townend, H. Whyte, and A. T. Woolward. The St. John Ambulance Association will supply a full staff of orderlies. The institution will probably be known as Queen Mary's Hospital, and Lieutenant-Colonel Openshaw will be in command. He was in the Boer War with the Imperial Yeomanry Hospital, and has therefore, like Mr. Mosely, had practical experience of the problems which he left on Wednesday night to face. We have high authority for stating that the scheme has the approval of Sir Arthur May, Director-General of the Navy, and that the lines on which it will develop have been mapped out in consultation with him.

### PANIC OFFERS OF PRIVATE HOUSES.

The number of offers by private individuals to place their land, houses, and buildings at the disposal of the Government or the Red Cross Society for convalescent institutions or hospitals threatens to become embarrassing. To record the state of feeling prevalent, however, we add to a list elsewhere the following offers. The Colonial Secretary, Mr. Harcourt, has offered his home, Nuneham Park, as a convalescent hospital to the Red Cross Society, which, it is understood, is also in a position to make use of Welbeck Abbey, the home of the Duke of Portland, and Grosvenor House and Eaton Hall, the town and country houses of the Duke of Westminster, for hospital purposes. The result is that the British Red Cross Society has been compelled to announce that the offers of hospital accommodation from private sources are likely to be far in excess of the anticipated demand; and that, in fact, gifts of money and subscriptions would be far more useful than any offers in kind at the present time.

### A POST WHICH NO ONE IS ASKED TO FILL.

A moment's thought will show the truth of this, for while the offer and acceptance of private hospital accommodation necessarily involves an enormous amount of organisation, adaptation, and management, which at any time would be difficult to cope with on the spur of the moment, cash payments present no such difficulty. The tendency to panic in many of the offers received show that far too many donors forget that not only hospital buildings and medical staffs are needed, but also hospital managers, and men who have had some experience in a hospital secretary's office, and yet, so far as we are aware, there has not been a single request for a hospital manager from any quarter. Patriotism and necessity apart, many hospital secretaries will be glad to have no responsibility in a matter which involves providing order, administration, and control in a building empty of everything except the patriotic fervour of its donor.

### THE LONDON HOSPITAL STAFF WHO HAVE LEFT FOR THE FRONT.

We give below the names and numbers of the staff of the London Hospital who have volunteered for service in the present crisis. The following is a list of names of medical men in the service of the hospital who have been accepted, and have either left or are expected to leave daily. Apart from the whole of the staff of the Base hospital to be erected at South Queensferry by Mr. A. Mosely, whose names we note elsewhere, and who are, with one exception, from the London Hospital, the other names are as follows:—Drs. F. G. Chandler, A. E. Herman, J. Bostock, J. H. Pendered, H. W. Batchelor, A. R. MacMullen, A. D. Ball, H. Gwynn Jones, E. Phillips, L. D. Cohen, W. A. Stewart, C. J. G. Taylor, H. Wetherbee, and a large number of students from the Medical College. We are officially informed that it is possible, too, that there may be a further call by the Red Cross Society. As regards the kindred difficulty of the hospitals in relation to their contracts, in many cases hospital secretaries are in the midst of negotiations with their contractors, and at present detailed publication of the position would not in the institutions' interests be fair.

### DUCHESS OF WESTMINSTER'S WAR HOSPITAL.

Among the first rush of privately-volunteered hospitals which obtained the consent of the British Red Cross Society is that undertaken by the Duchess of Westminster. The approval of the Director-General of the Army Medical Service has been obtained, and the plan is to organise and equip a base hospital to be sent to the seat of war with the least possible delay. Mr. Gordon Watson has been one of the first to join the medical staff, and the finance of the scheme, apart from subscriptions which may be sent for it to Drummond's Bank, is to take the form of a gift of £1,000 from the Duchess of Westminster, who is guaranteeing £400 a month so long as the hospital is required. Those who watched carefully the hospital organisation excited by and required for the war in South Africa will remember that all the projects which were set on foot in the first moment of enthusiasm did not survive the test of practical politics, a test which revealed the weak points alike in individual volunteers, hospital ships, and private effort generally. At this time, however, it is our duty to record impartially the principal efforts made, but we reserve our opinion as to the practical value of all of them.

### LONDON HOSPITALS' OFFER TO WAR OFFICE AND ADMIRALTY.

The Great Northern Central Hospital, Holloway, has placed 100 beds at the disposal of the Government for the reception of soldiers and sailors wounded or invalided from active service during the war. Until such beds are needed the usual work of the hospital continues, and the authorities have made provision for the treatment of all accidents or urgent cases among the poor of North London, in the event of the beds being required by the Government. Simultaneously the board of management of the London Homœopathic Hospital, Great Ormond Street, W.C., has placed a number of the 163 beds in the hospital at the disposal of the Director-General of the Admiralty, which have been accepted with cordial thanks. We understand that a number have also been placed at the disposal of the military authorities. The casualty department and certain wards have been reserved for the general use of the hospital. The board of management of the Metropolitan Ear, Nose, and Throat Hospital have also decided to offer the service of their institution to the Admiralty and the War Office, the staff having unanimously offered their services for the care of war patients.

### HOSPITAL PREPARATIONS BY PRIVATE GENEROSITY.

Among the numerous offers of private houses and other buildings for use or adaptation to hospital purposes the following may be mentioned as among the first batch received by the Admiralty and the War Office: The Grand Duke Michael of Russia, Lord Spencer, Lord Onslow, Lord Sefton, Lord Halifax, Lord Chetwynd, Lord Northcliffe, who has placed Elmswood, Broadstairs, at the disposal of the Government, Lord Desborough, the Hon. Lady Miller of Manderston, Mr. Humphreys Owen, Mr. A. W. Tate, Mrs. Claude Watney, Mrs. Irene Osgood, the novelist, of Northampton. But in addition to these must be mentioned the offers of several Oxford Colleges, including Christ Church, Balliol, New College, and Keble, while the Church Army, the Fishmongers' Company, and the British Portland Cement Company have also made proposals of a similar kind. Lord Bute has also offered Cardiff Castle for military purposes, and the Duke of Sutherland has offered Dunrobin as a surgical base for the North Sea Fleet.

### HOSPITAL ADVICE ON THE CONVERSION OF HOUSES.

Following his own example in placing Dunrobin Castle, Sutherlandshire, at the disposal of the Government as a hospital, the Duke of Sutherland suggests that many like himself would be willing to lend their country houses on the East and South-East Coasts, and near London, for hospital purposes. As to the practical details of converting them, he writes: "For the purposes of converting the houses into hospitals and convalescent homes expert advice is advisable, and will those who are prepared to offer their houses on these lines write to me, with full particulars of their houses and accommodation, at 25 Victoria Street, London, S.W., where I have made arrangements for all inquiries to be attended to by those who are fully competent to do so? I feel certain that a central bureau of this description would considerably facilitate the medical departments of the Admiralty and of the War Office, and enable them to get a list of people willing to lend their houses at the shortest notice, and, in addition, enable those who wish to help in this way to do so in the most efficient manner." Viscount Halifax has placed Hickleton Hall, his Yorkshire seat, at the disposal of the Government for convalescent officers. Viscount Chetwynd's residence, Wyndthorpe, near Doncaster, is to be open to the wounded. The Doncaster Corporation have arranged to turn the Mansion House into a hospital.

### THE QUEEN AND HOSPITAL LINEN GUILDS.

The ladies' leagues and linen guilds throughout the country connected with the voluntary hospitals will read with interest an appeal issued by Queen Mary, from which we extract the following: "I appeal to all the presidents of the needlework guilds throughout the British Isles to organise a large collection of garments for those who will suffer on account of the war, and I appeal to all women who are in a position to do so to aid the guilds with their work. Garments will be of service to the soldiers, sailors, and Territorials, to their families, to the military and naval hospitals, and to those among the poorer classes of the population who will suffer from any distress that may arise. I hope that the guilds will co-operate with the Prince of Wales's National Relief Fund and with the Red Cross Society, who have the organising of working parties among their schemes. . . . The most useful garments for soldiers and sailors on active service are flannel shirts, socks, sweaters, and cardigan jackets; for the naval and military hospitals, nightshirts, pyjamas, flannel bed-jackets, and bed-socks, which would be distributed by the British Red Cross Society. . . . I have arranged that all work by my guilds in London shall be received at Friary Court, St. James's Palace, and distributed from there to the various associations. I trust that the presidents of the guilds all over the country will follow my example, and co-operate with the local officers of the same organisations and arrange with them for the local distribution of the garments. It should be remembered that all flannel garments should be made on a large size, and suitable paper patterns can be obtained from Butterick, 175 Regent Street. Anyone who is willing to assist in this work can obtain on application the name and address of the president of their local needlework guild. All letters and parcels should be addressed to the hon. secretary, Friary Court, St. James's Palace, S.W., and should be marked Q.M.N.G.—Mary R." As probably the best organised guilds of this class throughout the country are those in connection with hospitals, the Queen's wishes in this matter will be especially interesting to them.

### CONVALESCENT HOMES FOR WOUNDED SAILORS.

Princess Christian, the president, and Lord Cheylesmore, the chairman, of the Incorporated Soldiers' and Sailors' Help Society, point out that it is the official duty of the Incorporated Soldiers' and Sailors' Help Society in time of war to organise temporary convalescent homes for wounded and disabled sailors and soldiers, and therefore that they will be glad to receive formal notice from all those throughout the kingdom who are in a position to place homes or beds at their disposal for this purpose, stating nearest railway station, distance therefrom, and accommodation available. Representatives of the Society everywhere are asked to ascertain the accommodation in their immediate neighbourhood, and report at once to Major Tudor Craig, at the Society's address, 122 Brompton Road, London, S.W.

### COTTAGE HOSPITALS AND THE WOUNDED.

Last week the committee of the Hornsey Cottage Hospital met and considered the question of forming a war hospital for Hornsey for soldiers and sailors. It is proposed to erect temporary buildings in the hospital grounds. Several cricket clubs, whose grounds adjoin those of the hospital, have offered the use of their premises. The medical staff, here as elsewhere, have eagerly proffered their services, as also have a number of nurses.

### THE PATRIOTISM OF THE HULL GUARDIANS.

We published particulars in our issue of July 25 of the new Poor-Law Infirmary at Kingston-upon-Hull, which was opened by Mr. Hargreaves, the Lord Mayor. As we are now informed that the Guardians have placed their new institution at the disposal of the Government, in view of the war, and of the strain which may be put at any moment on hospital accommodation of the East Coast, we think it worth while republishing the principal points about the new institution, showing how valuable and patriotic the Guardians' offer may prove to be. The building provides accommodation for 236 beds. It is four storeys in height. In the centre is the administrative block, on either side of which are the wards. On the first three floors there are two large wards, 96 feet long by 24 wide, containing thirty-one beds apiece, and two small isolation wards. The fourth floor is meant for phthisical cases. Half of it is occupied by cubicles and the rest of the space is open, so that the beds can be wheeled out upon it. Access to each floor is gained by a staircase with a lift, large enough to take a bed, in the centre. The building is heated by a forced circulation, high-pressure system, and is lighted by electricity. A novel feature, by the way, is the provision of a vacuum cleaner on each floor. It is at once obvious that the offer of a new institution of this size is of great importance, and not only falls into a category of its own, but sets an example to other Guardians, especially those on the East Coast.

### EFFECTS OF THE WAR ON PRIVATE PRACTICE.

Owing to the liberal offer of the Government of 24s. a day to civilian surgeons, which we noted in our last issue, and in addition to the other causes which have been acting during the last two years, the supply of *locum tenentes* at the present moment has fallen to an unprecedentedly low limit. This scarcity is likely to become a serious matter for many practitioners who belong to the R.A.M.C. (T.F.). In addition to the sacrifices they are making in serving the country, they stand to suffer great pecuniary loss in their practices at home. The matter is also becoming somewhat disconcerting for many hospitals and infirmaries, which have found it increasingly difficult to obtain residents, as our readers are aware, and now we hear that the resident staffs of numerous hospitals, for instance in London, are reduced to almost below the efficient minimum. It is a pleasure to learn that in many districts, for instance in Southampton, meetings of the general practitioners have been held and measures have been taken by those doctors who are not leaving for service to safeguard loyally the interests of their colleagues and to undertake any medical services for the sole benefit of the absentees. If such a spirit of fraternity and camaraderie becomes general it will go far to ensure adequate treatment of the population and to minimise the loss to those called to duty. In the event of great necessity it is unlikely that the large band of young medical men engaged in school inspection and tuberculosis work will feel called upon to continue their duties, which after all could easily be shared out for some months without entailing too great disadvantage to the community. The country existed somehow without them only about five years ago. In this way it may be confidently hoped that any dire suffering among the sick will be obviated, and assistance will be forthcoming for the overworked house surgeons and resident medical officers in our institutions.

### THE CRYSTAL PALACE AS A HOSPITAL.

On Monday last the purchase of the Crystal Palace was completed with the payment of the purchase money—£230,000—to Lord Plymouth.

Now vested in the trustees, with Sir David Burnett as chairman and Mr. J. E. W. Robinson as clerk, the palace and grounds are for the use of the people. An executive committee of thirty-two members has been appointed, and Sir David Burnett has announced that he has offered the grounds and buildings to the War Office as a temporary hospital.

### THE FIRST ACCEPTED HOSPITAL SHIPS.

We are able in this issue to mention that Lord Tredegar's steam yacht, the offer of which we announced last week, has been accepted by the Admiralty for service as a hospital ship. The yacht, which is christened *Liberty*, is a vessel of 1,600 tons. The staff on board will include several well-known surgeons from Guy's Hospital, together with a complete medical equipment. The whole expense has been borne by Lord Tredegar, who has been made a lieutenant in the Royal Naval Reserve. At the same time the Duchess of Connaught has telegraphed to Mr. Churchill that the women of Canada are anxious to offer a hospital ship to the Mother-country, and asking whether, before a fund be opened, the offer would be acceptable to the Admiralty. Mr. Churchill has replied accepting, on behalf of this country, the loyal offer with the warmest thanks. In this connection we may draw attention to Lady Sarah Wilson's appeal for the organisation and equipment of "a base or stationary hospital for English, French, and Belgian soldiers, to be established on the Continent, the locality of which would be determined by the progress of hostilities." The relation of this proposal to the above offers of hospital ships becomes clear from Lady Sarah Wilson's recollection of the South African War, of which she says: "I am haunted day and night by the idea of those grievously wounded having to face additional discomforts in the shape of a crowded ship, a rough sea." "It would be an inestimable boon," she continues, "for the Army base hospitals to have some cases—perhaps the most severe ones—taken off their hands." "I propose," she concludes, "to form a small committee, and to run the hospital under the auspices of the Red Cross Society." Subscriptions should be sent to 16 Grosvenor Street. What are our readers' views?

### THE STIGMA ON BIRMINGHAM ATTENDANTS.

As we expected, the remark of Councillor Davis, Chairman of the Asylums Committee, when presenting a supplemental report to the Birmingham City Council, to the effect that "the nurses and attendants were an utterly unskilled class," has excited controversy. An odd turn is given to this by an "attendant," who remarks that "we should hardly have expected the phrase to have come otherwise than from within" a mental hospital. As a matter of fact the phrase is absurd, for though all acquainted with mental hospital administration are aware that it is often difficult to procure the right type of attendant, and particularly of nurses, at the present time the standard alike of work and pay is improving. It is impossible to remain for three years, as they are generally bound to do, in a modern mental hospital, and pass a medico-psychological examination, and to be "utterly unskilled." Councillor Davis should explain what he meant, and produce the evidence on which he based this sweeping and distorted assertion.

### END OF THE BLACKPOOL HOSPITAL DEADLOCK: THE BOARD RESIGN.

We are glad to announce that an end has been put at last to the Blackpool Victoria Hospital deadlock, and, as we foresaw in these columns from the first, a necessary first step to a settlement has been the resignation in a body of the board of management. On their resignation the management became immediately vested in the trustees, Alderman J. Bickerstaffe, chairman of the defunct board, Mr. T. Loftos, secretary to it, Dr. Butcher, and Mr. Coop. The mediation committee, consisting of Messrs. Broadhead, J. Collins, and M. G. Wilde, fired no doubt by this return to sanity, continued its already protracted deliberations with eight representatives of the alienated local medical men at Dr. Dunderdale's house. Virtually the doctors have won all along the line, and in view of the facts, of which our readers have been regularly informed during the past seven months or so, they will agree that this victory was inevitable and is a just vindication of the main points in their case. By resigning *en bloc* the board *ipso facto* ended the dispute. The board is now to be reconstructed, and that means that the narrow clique which took arbitrary sides with the officers it chose to support and those it chose to condemn has been routed, and a more representative board is to take its place. The medical men are to have four seats instead of two; the medical staff is to be chosen by the practitioners of the borough; the revision of the trust-deed is to follow on democratic lines, which we hope will put an end once for all to the voting powers previously absorbed by various companies and organisations. The boycott of the institution by the medical staff is, therefore, at an end, and it only remains for the general body of the subscribers and the public to unite on the opportunity which the local medical men have fought and at last won for them, so that this hospital, the mismanagement of which made it the disgrace and laughing-stock of the voluntary system, may become from the present moment a healthy institution, with subscribers, medical men, and committee all united in the one resolve that the hospital's welfare must take the first place, and the best care and treatment of the sick in it override all personal intrigues, differences, and ambitions.

### GUY'S HOSPITAL AND DEFECTIVE RECRUITS.

The whole of the large resources of the dental department at Guy's Hospital has been offered by the governors of the hospital to the War Office for the dental treatment of would-be recruits who have been rejected on the ground of defective teeth. The treatment will be undertaken principally by old Guy's men at present working in the Metropolitan area, who will work in relays.

### BRISTOL ROYAL INFIRMARY AND THE WAR.

The Bristol Branch of the Red Cross Society is responsible for the provision of 500 beds, and in 1911 the governors of the Royal Infirmary generously promised in the event of necessity to place their whole establishment and Nurses' Home at the disposal of the Government. We are informed by a correspondent that, in view of the present crisis, preparations are being made to establish that institution as a base hospital, and the authorities are endeavouring to secure the co-operation of other institutions and nursing homes for the reception of their patients; some will be sent to the Royal Mineral Water Hospital, Bath, the remainder of beds in the institution having been placed at the disposal of the country.

### OVERCROWDING AT A MANCHESTER INFIRMARY

The house committee of the Withington Workhouse Hospital under the South Manchester Board of Guardians were complaining, on the brink of the present war, that the overcrowding which has been admitted, even after temporary arrangements had been come to, would leave the staff no margin of beds for the winter. The Local Government Board has already approved the provision of an additional pavilion on a site known as that adjoining number 9. It has been decided to proceed with this and with the children's hospital, the plans of which are receiving the attention of the Local Government Board. We comment on these proposals not only because overcrowding is an evil which must be denounced and put an end to wherever it arises in institutions for the sick, but also because the present war does not do away with the need for prompt action, however much it may distract our attention from it. The announcements which have been made, that the Government offices and the local authorities intend to prosecute as many building and other schemes as possible in order to reduce unemployment, we hope will seriously be acted upon. There is always a risk that abuses like the above may take a new lease of life at a time when a European war is absorbing everybody's attention. But those who read our leading article on "Poor-Law Infirmaries and the War," and the letter which we publish on the same subject from an infirmary officer, with the suggestion which he makes, will realise that, in fact, among all workhouse hospitals and infirmaries of any size and claim to a high standard the overcrowding problem may become actually more acute, and will need at any rate our best immediate attention to prevent and to forestall.

### WOMEN'S COMPETITION FOR SECRETARIAL POST.

The recent opening by Princess Henry of Battenberg at Portsmouth of the new wing at the South Hants Eye and Ear Infirmary reminded the public that this is one of the few institutions which possesses, and has possessed for some years now, a woman hospital secretary. Miss Julia Beckett, the holder of this post, is one of the handful of her sex in this position, and though women resident medical officers are to be found outside the two or three hospitals staffed entirely by them, and in Poor-Law infirmaries here and there, Miss Beckett's institution is on the way to becoming something more than the small special hospital which it has been in the past. The fact is worth mentioning in view of the proper attitude, as a specialty, with which hospital secretaries regard their profession. Understandably jealous of medical superintendents, whom they feel to be competing with a professional medical training unfairly with a professional business one, in a post where it is more important for a man to be a good organiser than to be medically qualified, the competition of the woman hospital secretary has hardly yet made itself sufficiently felt to evoke more than passing comment. But we cannot shut our eyes to the fact that if women's hospitals continue successfully to place their business affairs in the hands of women and find their administration to their liking, competition, however slowly, in this branch of hospital life, as in medicine, is bound one day to come. It was really as much in her capacity of secretary and organiser as in that of matron and trainer of nurses that Florence Nightingale showed her powers. What effect would women's competition for secretarial posts have not only on the quality of the work, but on the salaries? A large part of the energetic modern secretary's work is unpaid in the sense that the same keenness and ability would command a higher reward in the world of commerce. What would be the effect and what are the chances of women entering into the secretarial profession in larger numbers? We invite our readers to forecast the result.

### THE INFECTION OF NON-PHTHISICAL PATIENTS.

In view of an article, which at the last moment has been crowded out, on "Tuberculosis Pavilions at Isolation Hospitals," the recent statement reported as being made by the clerk to the South Manchester Guardians, that "we have well-authenticated cases of patients who have contracted phthisis when in our hospital wards through the mixing of phthisical with non-phthisical patients," is interesting. The article discusses the possibility of risk, under proper conditions, between phthisical patients in a special block, and the rest of the patients and the staff on the site of a hospital containing it, and the discussion of the evidence is convincing, as our readers will see, we hope, next week. We trust that the clerk to the South Manchester Guardians may realise the importance of making the evidence, upon which his reported remark was made, available, for until it is forthcoming the inference among impartial and experienced sanatorium superintendents and tuberculosis officers will be either that he has jumped to conclusions which examination will not warrant, or else that the conditions which obtain in the institution where the alleged infection has occurred must be the reverse of adequate and proper.

### THE ENDING OF THE BARRY HOSPITAL DISPUTE.

The resignation of the voluntary staff at the Barry Accident Hospital, Glamorganshire, which we noted in this column on August 1, on account of the committee's proposal to appoint a resident medical officer at a salary of £600 a year, has apparently at last been settled by a scheme which the District Council, who control the hospital, has now adopted. Mr. F. E. J. Murrell, chairman of the hospital committee, has put forward, with the approval of Dr. G. Neale, the local medical officer of health, seven proposals which may be summarised as follows:—That Dr. Neale be appointed medical superintendent of the infectious diseases hospital at a salary of £150, and retain the office of medical officer of health for the port sanitary authority. Further, that Dr. Kent be appointed in place of Dr. Neale, who will resign, at a salary of £100 per annum. He shall be responsible for supervising and superintending the health department and school clinic, and shall have charge of the accident hospital. Thirdly, that two assistants be appointed, one as assistant school medical officer, at a salary of £300 per annum, rising by two annual increments to £350. Fourthly, a rota of three local surgeons to be selected by the council, who can be called upon to operate as needed. The council to give an honorarium of £100 per annum as a retaining fee. Fifthly, that local practitioners may follow their cases into the hospital, and operate with the consent of Dr. Kent. He may also engage a specialist for operations at the hospital on the same condition, free of cost to the council. Sixthly, all officers in the departments to be under the medical officer of health. And, lastly, on the retirement of Dr. Neale in November 1915, Dr. Kent be offered the port sanitary work and charge of the infectious diseases hospital, at the extra remuneration of £100 per annum, making a total salary of £600 per annum. The scheme was unanimously adopted, and Dr. E. J. H. Budge, on behalf of the local medical gentlemen, expressed the opinion that as far as the doctors were concerned there would be no difficulty.

### THE FINAL SOLUTION OF WORKHOUSE NURSING

We dealt last week in our second leading article with the main faults from an administrative point of view of the Nursing Order. Supporting our general case with further details, we may note that other instances where the Order is at fault are the provision which makes it incumbent upon the midwife to inform the master, even when there is a resident medical officer, if she requires the attendance of a registered medical practitioner in accordance with the rules of the Central Midwives Board, and the section which empowers the master on his own initiative to notify the relatives that an inmate is seriously ill. Lastly, as regards the classification of sick children, the recent deputation does not seem to realise the difficulties of separating completely adults from children in a hospital which admits both. The admission of suckling infants with their mothers, of children who have been exposed to the infectious diseases of childhood, of phthisical children, and of adults suffering from such diseases as measles, whooping-cough, etc., renders it impossible in practice to separate completely the two classes. There is probably no general hospital in England where a rigid classification of this kind is or could be carried out. And the difficulties are greater of the Poor-Law infirmary, because it has to admit any form of contagion from patients of infectious diseases which are excluded from the general hospitals. If the Local Government Board had possessed a nursing service we may reasonably conclude that the head of the service would have been in a position to prevent the grave mistakes in nursing matters made by the Board in the Poor-Law Institutions Order. Finally, can there be any doubt that the only way to meet the problem of the nursing of the smaller workhouses is to staff them from a service the members of which can look upon such posts as parts of a graded and organised system, leading to promotion to higher and more responsible positions?

### THE DOCTORS' WIVES ASSOCIATION.

We are requested to state that the present necessity for practising economy may make it a matter of interest to doctors' wives who are members of this Association to know that, besides the saving of expense in the purchase of goods now open to them, special arrangements have been made for the supply of flannel, pins, needles, scissors, cotton wool, and bandages, a fact which those interested may like to hear of. The saving of expense on practically most of the articles of ordinary requirement, *except food*, will be found material. Officers outfitting and officers receiving commissions will find the Association's aid valuable by effecting an appreciable saving. Economy can be secured in the purchase of Gamgee lint, cotton-reeled, sewing machines, for charitable work. The Association has special means of supplying members often on special terms. All communications should be addressed to the Secretary of the Association, 28 Southampton Street, Strand, London, W.C.

### GERMANY AND THE DRUG MARKET.

The Drug market remains in an uncertain condition, although with the exception of such drugs as are obtained from Germany the advances in prices have not on the whole been greater than might have been expected. Such products as phenacetin, sulphonal, and other synthetic drugs cannot be replaced when the supplies at present in the country are exhausted. Salicylates, bromides, iodides, carbolic acid, glycerine, opium, etc., are among the drugs which are also affected to a marked extent; while bandages and other surgical dressings are substantially dearer. The Secretary of the Pharmaceutical Society last week addressed a communication to the Secretaries of the Pharmaceutical Committees throughout the country, requesting them to point out to the clerks to their Insurance Committees that many drugs in common use are made abroad under foreign patents and that others are manufactured from raw material that does not exist in this country; the communication referred to the necessity of husbanding the supply of drugs in the country.

